# Levels of Serum 25-Hydroxy Vitamin D in Benign and Malignant Breast Disease Patients: An Observational Study

**DOI:** 10.7759/cureus.78283

**Published:** 2025-01-31

**Authors:** Deepak Soni, Amit Tiwari, Priya S Kushwah, Shruti Dubey, Shehtaj Khan

**Affiliations:** 1 Surgery, Hind Institute of Medical Sciences, Barabanki, IND; 2 General Surgery, LN Medical College and Research Center, Bhopal, IND; 3 Emergency Medicine, LN Medical College and Research Center, Bhopal, IND

**Keywords:** 25-hydroxy vitamin d, benign breast diseases, breast cancer, post-menopause, vitamin d

## Abstract

Introduction: Vitamin D deficiency is the foremost underdiagnosed and under-treated nutritional deficiency in the world. This vitamin deficiency has reached pandemic proportions despite being synthesized in the human body under sun exposure. Vitamin D plays a significant role in the maintenance of reproductive health and physiology in the human body. Its deficiency has been studied extensively in correlation with breast diseases and has been implicated as a risk factor for breast cancer by many. Thus, we undertook this study on patients with benign or malignant breast disease to assess the levels of vitamin D in the blood.

Methods: This prospective observational study was conducted at a tertiary care center in central India. All adult female patients who were admitted to the Department of Surgery with breast disease from January 2023 to June 2024 were included in the study. Exclusion criteria were women who had taken vitamin D supplementation in the previous two years, those with pre-existing vitamin D deficiency, or those who took therapy for osteopenia or osteoporosis. Patients finally included in the study were tested for serum vitamin D levels, estimated by the chemiluminescence method.

Results: Out of 85 patients with benign or malignant breast diseases, 37 (43.5%) patients had normal vitamin D levels, 34 (40.0%) had vitamin D insufficiency, and 14 (16.5 %) had vitamin D deficiency. Sixty-nine (81.17%) patients had benign breast disease and 16 (18.83%) had breast cancer. As compared with benign breast disease patients, those with malignant breast disease had significantly low serum 25-hydroxy vitamin D levels (p-value <0.001).

Conclusion: The significant difference in mean 25-hydroxy vitamin D levels between benign breast disease and breast carcinoma patients favors the recommendation that vitamin D supplementation may eliminate a potential risk factor in breast cancer development, though larger sized studies and randomized trials are needed to establish a definite clinical correlation. While considering vitamin D supplementation, its non-skeletal effects should be taken into account more than the skeletal effects.

## Introduction

Vitamin D deficiency is widespread throughout the world, yet it is the foremost underdiagnosed and under-treated nutritional deficiency in the world [1]. The synthesis of vitamin D occurs in the skin under sun exposure mainly through ultraviolet B radiation of wavelength 290-320 nm [2]. India may be a country with ample sunshine, but Indian socio-religious and cultural practices do not facilitate adequate sun exposure [3]. Also, most of the population in India has a vegetarian diet but the common sources of vitamin D are of animal origin. Even the milk supplied in India is not vitamin D fortified. Vitamin D plays an important role in the reproductive health of women by regulating various hormones like estrogen, progesterone, human chorionic gonadotropin, and human placental lactogen [4]. Calcitriol, the active metabolite of vitamin D, binds to vitamin D binding protein and reaches its target tissues to exert its endocrine functions through the vitamin D receptor (VDR) [5]. However, vitamin D may also induce VDR-independent effects [6]. This vitamin has been shown to play a very important role in the development and performance of the mammary gland and brings about its effects in multiple ways. Calcitriol, 1,25-(OH)2D, suppresses the aromatase gene and thus decreases estrogen production. ERα, the nuclear receptor that mediates estrogen actions, is also downregulated by calcitriol [7,8]. Calcitriol, thus, reduces the amount of estrogenic hormones as well as their biological activity in the breast [9]. Also, the BRCA1 gene (breast cancer 1) is induced by vitamin D and is inversely correlated with cell proliferation [10]. Together with growth arrest, it may induce apoptosis in carcinoma cells, as MCF-7 (Michigan Cancer Foundation-7) cells treated with this hormone show cell shrinkage, chromatin condensation, and DNA fragmentation [11]. Vitamin D deficiency has been studied extensively in correlation with breast diseases, but the links remain weak. We hypothesize that patients with malignant breast disease have significantly lower vitamin D levels compared to those with benign breast disease. Proving this could have clinical significance and help us devise a preventive strategy in the form of vitamin D supplementation. Thus, we undertook this study on patients with benign or malignant breast disease to assess the levels of vitamin D in the blood.

## Materials and methods

This study was conducted prospectively at LN Medical College, Bhopal, located in central India. All adult female patients who were admitted to the Department of Surgery with breast disease from January 2023 to June 2024 were included in the study. Exclusion criteria were women who took vitamin D supplementation in the previous two years, those with pre-existing vitamin D deficiency, and those who took therapy for osteopenia or osteoporosis. Patients finally included in the study had their demographic data recorded and were tested for serum vitamin D levels, estimated by the chemiluminescence method in a CL-900i Mindray device (Mindray Medical India Private Limited, Gurugram, India) at our center, with daily quality checks and monthly EQAS. Lab values for serum levels of 25-hydroxy vitamin D were categorized as follows: normal: 30-100 ng/ml, vitamin D insufficiency: 20-30 ng/ml, and vitamin D deficiency: <20 ng/ml.

Statistical analysis

Data was represented in numbers, percentages, mean, and standard deviation. A p-value of <0.001 was considered significant. The study was approved by the Institutional Ethics Committee.

## Results

A total of 104 patients fulfilled the inclusion criteria, but 19 patients were excluded after applying the exclusion criteria, and 85 patients were finally enrolled in the study. The age of patients ranged from 18 to 62 years, with a mean age of 34.85 +/- 9.26 years. Age-wise distribution of vitamin D levels is shown in Table 1.

**Table 1:** Age-wise distribution of patients along with their mean vitamin D levels

Age of patients	No. of patients (percentage)	Average age (S.D.)	Average vitamin D level (S.D.)
18-25 years	13 (15.3%)	21.62+/-2.23	33.83+/-6.89
26-35 years	39 (45.9%)	31.72+/-2.95	30.59+/-4.68
36-45 years	20 (23.5%)	39+/-3.11	27.45+/-9.04
> 45 years	13 (15.3%)	51.08+/-4.54	15.12+/-4.73
Total	85 (100%)	34.85+/-9.26	27.98+/-8.58

Thirty-seven patients (43.5%) had normal vitamin D levels, 34 (40.0%) had vitamin D insufficiency, and 14 (16.5 %) had vitamin D deficiency. There was a sufficient mean vitamin D level in women </=35 years of age as compared to the women aged >35 years, who had vitamin D deficiency. Out of 85 patients, 21 (24.7%) patients were post-menopausal and 64 (75.3%) were pre-menopausal. In pre-menopausal women, the mean vitamin D level was 30.6 ng/ml, and in post-menopausal women, it was 24.3 ng/ml. The mean age and vitamin D level in patients with different diagnoses are shown in Table 2.

**Table 2:** Mean age and vitamin D levels in patients with different diagnoses The table shows the distribution of patients according to their diagnosis and their respective mean vitamin D levels. S.D.: Standard deviation

Diagnosis	No. of patients (percentage %)	Average vitamin D level +/- S.D.	Mean age +/- S.D.
Fibroadenoma	50 (58.8 %)	30.85 (+/-5.25)	30.58 (+/-6.58)
Carcinoma of the breast	16 (18.8%)	13.86 (+/-3.580)	49.4 (+/-5.34)
Breast abscess	14 (16.5%)	32.77 (+/-6.89)	33.14 (+/-3.87)
Fibroadenosis	4 (4.7 %)	31.5 (+/-3.10)	33 (+/-3.46)
Phylloid tumor of the left breast	1 (1.2%))	29.5	45
Total	85 (100%)	27.98 (+/-8.58)	34.85 (+/-9.26)

Out of 69 (81.17%) patients with benign breast disease, 37 (53.62 %) patients had normal vitamin D levels, and 32 (46.38%) patients had insufficiency. Out of 16 (18.83%) patients with carcinoma of the breast, 14 (87.5 %) patients had vitamin D deficiency, and two (12.5 %) patients had insufficiency. As compared to the benign breast disease patients, those with malignant breast disease had significantly low serum 25-hydroxy vitamin D levels (p-value < 0.001).

## Discussion

This study assessed the serum vitamin D levels in benign breast disease and breast carcinoma patients. In our study, patient's ages ranged from 18 to 62 years with a mean age of 34.85+/-9.26 years. The mean vitamin D level was 27.98 +/- 8.58 ng/ml, which falls within the insufficiency category. An inverse correlation was found between age and vitamin D levels, which is in accordance with the study conducted by Alipour et al. in 2014 [12]. Forty-eight (56.5 %) of our patients had low vitamin D levels. Vitamin D deficiency ranges from 40% to 99% [13]. This indicates that our study population had a lower prevalence of vitamin D deficiency as compared to that reported in various studies. In our study, 90.5% of post-menopausal women had vitamin D deficiency, which is in line with the reported vitamin D deficiency in post-menopausal women ranging from 50% to 90% in India [14]. Out of 68 patients with benign breast disease (the average vitamin D level was 31.6+/-5.55 ng/ml), 37 patients (53.62 %) had normal vitamin D levels, and 32 patients (46.38 %) had insufficiency. According to Bharthiraja et al. (2018), 64% of benign breast disease patients had normal vitamin D levels, 28% had insufficiency and 8% had vitamin D deficiency [15]. According to our observations in breast abscess patients, the mean vitamin D level was 32.77+/-6.89 ng/ml, which is contradictory to that reported by Lippolis et al. who demonstrated a low level of serum vitamin D as a risk factor for a few infectious diseases (mastitis) [16]. Of the 16 women with a diagnosis of carcinoma of the breast (mean vitamin D level: 13.86 +/-5.34 ng/ml), 14 patients (87.5 %) had calciferol deficiency, and a couple of patients (12.5 %) had insufficiency. Studies have attempted to highlight the importance of vitamin D in breast cancer. Voutsadakis et al., in a meta-analysis, researched vitamin D levels in patients with newly diagnosed breast cancer [17]. They found mean vitamin D levels in study patients to be 26.88 ng/mL, while in control patients, it was 31.41 ng/mL, relating vitamin D insufficiency to breast cancer. Shaukat et al. supported these findings while studying the relationship between vitamin D deficiency and the risk of developing breast cancer [17,18]. In contrast, there are also numerous studies that found no correlation between Vitamin D levels and breast cancer [19-21]. A recent review article attempted to address this discrepancy. Torres and colleagues found that vitamin D ≥ 40.26 ng/ml ± 14.19 mg/ml could be protective against breast carcinoma. They concluded that dietary supplementation may help in achieving optimal vitamin D levels [22].

Limitations of the study

This was an observational study with a small sample size, no randomization, and lack of follow-up of the patients. There might be some potential bias due to the exclusion of patients who had taken vitamin D supplementation in the previous two years. Also, BMI, sun exposure, and dietary habits may be confounding factors in the study. Future studies might provide further evidence and overcome these limitations by taking larger randomized cohorts.

Despite the limitations, we can agree that interventional possibilities to extend vitamin D status (moderate sun exposure and supplementation) for a possible role of calciferol in cancer prevention and recurrence should be taken up with high priority in both epidemiologic and intervention studies.

## Conclusions

The mean serum vitamin D level in breast carcinoma patients was significantly low when put next to the benign breast disease patients. This finding supports the recommendation that vitamin D supplementation may be the key to eliminate a potential risk factor in breast cancer development. Despite the limitations like a small sample size, taking into account various previous similar studies, it can be safely concluded that vitamin D plays a significant role in breast health. This can be a guide for further studies of the interventional type where vitamin D supplementation can be administered and effects on breast health can be studied. The non-skeletal effects of vitamin D are also more important than the skeletal effects and may be considered when evaluating these supplements.
